# Molecular Pathogenesis of *BCR-ABL*-Negative Atypical Chronic Myeloid Leukemia

**DOI:** 10.3389/fonc.2021.756348

**Published:** 2021-11-11

**Authors:** Diletta Fontana, Carlo Gambacorti-Passerini, Rocco Piazza

**Affiliations:** ^1^ Department of Medicine and Surgery, University of Milano-Bicocca, Monza, Italy; ^2^ Hematology and Clinical Research Unit, San Gerardo Hospital, Monza, Italy; ^3^ Bicocca Bioinformatics, Biostatistics and Bioimaging Centre (B4), University of Milano-Bicocca, Milan, Italy

**Keywords:** aCML, SETBP1, ETNK1, BCR-ABL1-negative, molecular pathogenesis, MDS/MPN

## Abstract

Atypical chronic myeloid leukemia is a rare disease whose pathogenesis has long been debated. It currently belongs to the group of myelodysplastic/myeloproliferative disorders. In this review, an overview on the current knowledge about diagnosis, prognosis, and genetics is presented, with a major focus on the recent molecular findings. We describe here the molecular pathogenesis of the disease, focusing on the mechanisms of action of the main mutations as well as on gene expression profiling. We also present the treatment options focusing on emerging targeted therapies.

## Introduction

Atypical chronic myeloid leukemia (aCML), *BCR-ABL1*-negative, is a rare hematological malignancy belonging to the overlap category of myelodysplastic/myeloproliferative neoplasms (MDS/MPN) of the WHO classification of myeloid neoplasms (1). The MDS/MPN category was introduced in the third edition of the WHO Classification of Tumors (2) and includes myeloid neoplasms that exhibit, at presentation, laboratory, clinical, and morphologic features that overlap between myelodysplastic syndromes (MDS) and myeloproliferative neoplasms (MPN) (3).

Initially, aCML was described as an atypical form of chronic myeloid leukemia (CML) *BCR-ABL1*-positive, since at the onset it presents with many features of classical CML (4–6). In 2008, in order to incorporate new laboratorial as well as clinical information to refine diagnostic criteria for previously described neoplasms and to introduce newly recognized disease entities, the WHO classification was updated and published as part of the fourth edition of the WHO monograph series (7, 8). In this classification, aCML has been modified to aCML *BCR-ABL1*-negative to highlight that it was not merely a variant of classical CML (9). Finally, in 2016, after 8 years since the previous revision and owing to the massive amount of information generated by the new sequencing technologies, a revision of the 2008 WHO classification of hematopoietic neoplasms (1) was deemed necessary.

## Characteristics of aCML at Presentation, Diagnosis, and Differential Diagnosis

aCML is a disorder of the elderly, since it typically affects patients with an age ranging between 60 and 76 years (10). Even though the first reports about aCML suggested a female predominance (11) or no sex predominance (12), in more recent years, reports analyzing larger cohorts of patients suggest a slight male predominance (13–15), though the biological reasons underlying aCML gender preference are poorly understood. Its estimated incidence is 1 out of 100 cases of t(9;22), *BCR-ABL1-*positive CML, meaning approximately 1 case per 1,000,000 persons per year (4, 11, 16, 17).

From a diagnostic point of view, aCML is a challenging myeloid malignancy characterized by features belonging to both myeloproliferative and myelodysplastic syndromes. As its clinical features may overlap with other myeloid malignancies, its diagnosis still relies primarily on morphological criteria, e.g., the evidence of dysgranulopoiesis in bone marrow or peripheral blood. Typically, aCML patients present clinical features similar to *BCR-ABL1*-positive CML including splenomegaly and a neutrophilic leukocytosis; they also show prominent granulocytic dysplasia (e.g., hypogranular and hypolobated neutrophils, abnormal chromatin clumping, and pseudo-Pelger–Huet neutrophils) (9). The white blood cell (WBC) count is >13 × 10^9^/L, with ≥10% of immature granulocytes and <20% blasts in the peripheral blood (PB) and in the bone marrow (BM) (1, 9, 18). Due to the high WBC count, it is not uncommon to observe a monocyte count >1 × 10^9^/L, but the percentage of monocytes at onset is always lower than 10% of the total leukocytes, which is critical to discriminate between aCML and a closely related MDS/MPN disorder, known as chronic myelomonocytic leukemia (CMML). In contrast to CML, basophilia is not prominent as basophils represent <2% of all PB white cells (9, 19). A hypercellular BM with myeloid hyperplasia and prominent granulocytic dysplasia is a consistent feature; however, trilineage dysplasia may be present (3, 20, 21). The leukocyte alkaline phosphatase level may be low, normal, or increased, therefore lacking diagnostic utility (9, 19). The recent discovery of somatic mutations occurring in *SETBP1* (22) and *ETNK1* (23) in up to a third of aCML cases (22–24) led to the insertion of these mutations in the 2016 WHO revision as supporting criteria for the diagnosis of aCML (1). However, the presence of these mutated genes does not ensure a certain diagnosis (see below and **Table 1**).

**Table 1:** Mutational frequencies.

	aCML	*BCR-ABL1*-positive CML	AML	MDS	CMML	CNL	MDS/MPN-U
*SETBP1*	7.4–48 (13, 14, 22, 25)	0 (22, 26)	0 (22)	0 (22)	4–15 (13, 22, 25)	25–41 (13, 22)	10–16 (13)
*ASXL1*	20–81 (13, 14, 25)	9.7 (26)	6.5–20 (27–30)	15–24 (31, 32)	14–69 (13, 25, 28)	77 (13)	64 (13)
*N/K-RAS*	11–27 (13, 14, 25)	0 (26)	10–18.8 (29, 30)	0 - 5 (31)	3–48 (13, 25)	10 (13)	12 (13)
*ETNK1*	3.7–13.3 (13, 14, 23, 25)	0 (23, 26)	0 (23)	0 (23)	0–14 (13, 23, 25)	0–2.6 (13, 23)	0–4 (13, 23)
*SRSF2*	14–65 (13, 14, 25)	–	6.6 (30)	14 (31)	24–51 (13, 25, 31)	43.6 (13)	48 (13)
*EZH2*	19–30 (13, 14, 25)	0.6 (26)	2 (33)	5–8 (31)	7–10 (13, 25)	20.5 (13)	24 (13)
*RUNX1*	11–15 (13, 14, 25)	2.6 (26)	5–15 (29, 30, 34)	10–20 (31)	10–37 (13, 25)	2.6 (13)	4 (13)
*TET2*	27–33 (13, 14, 25, 35, 36)	0.9 (26)	9–23 (29, 30, 37)	20–25 (29, 31)	48–78 (13, 25, 29)	20.5 (13)	44 (13)
*CBL*	8–11 (13, 14, 25)	0–0.9 (26, 38)	0.9 (30)	10 (39)	5–19 (13, 25)	5 (13)	8 (13)
*CSF3R*	1–22 (13, 14, 25)	–	3 (40)	1.4 (40)	0–3 (13, 25)	50–80 (13)	4 (13)
*JAK2*	4–11 (13, 25)	1.5 (26)	0.9 (30)	16.7 (41)	2–3 (13, 25)	7.7 (13)	8 (13)

The mutational frequencies of the genes most frequently involved in the onset or clonal evolution of aCML, BCR-ABL1-positive CML, AML, MDS, CMML, CNL, and MDS/MPN-U are reported.

The differential diagnosis of aCML includes *BCR-ABL1*-positive CML, CMML, chronic neutrophilic leukemia (CNL), and prefibrotic primary myelofibrosis (pre-PMF) (**Table 2**). Besides the lack of the Philadelphia chromosome and the *BCR-ABL1* translocation, the major criteria that distinguish aCML from *BCR-ABL1*-positive CML are the dysgranulopoiesis, which is common in aCML but only occasionally reported in classical CML, and the normal basophil counts of the former (<2% of leukocytes) (1).

**Table 2:** Diagnostic criteria for *BCR-ABL1*-positive CML, CMML, CNL, and pre-PMF as defined by the WHO in the 2016 revision are listed.

**BCR-ABL1-positive CML, accelerated phase criteria**
Persistent or increasing WBC (>10 × 10^9^/L), unresponsive to therapy
Persistent or increasing splenomegaly, unresponsive to therapy
Persistent thrombocytosis (>1,000 × 10^9^/L), unresponsive to therapy
Persistent thrombocytopenia (<100 × 10^9^/L) unrelated to therapy
20% or more basophils in the PB
10%–19% blasts in the PB and/or BM
Additional clonal chromosomal abnormalities in Ph1 cells at diagnosis that include “major route” abnormalities (second Ph, trisomy 8, isochromosome 17q, trisomy 19), complex karyotype, or abnormalities of 3q26.2
Any new clonal chromosomal abnormality in Ph1 cells that occurs during therapy
**CMML diagnostic criteria**
Persistent PB monocytosis ≥1 × 10^9^/L, with monocytes accounting for ≥10% of the WBC count
Not meeting WHO criteria for BCR-ABL1-positive CML, PMF, PV, or ET
No evidence of PDGFRA, PDGFRB, or FGFR1 rearrangement or PCM1-JAK2 (should be specifically excluded in cases with eosinophilia)
<20% blasts in the blood and BM
Dysplasia in one or more myeloid lineages. If myelodysplasia is absent or minimal, the diagnosis of CMML may still be made if the other requirements are met and an acquired clonal cytogenetic or molecular genetic abnormality is present in hematopoietic cells or the monocytosis (as previously defined) has persisted for at least 3 months and all the other causes of monocytosis have been excluded
**CNL diagnostic criteria**
PB WBC ≥25 × 10^9^/L Segmented neutrophils plus band forms ≥80% of WBCs Neutrophil precursors (promyelocytes, myelocytes, and metamyelocytes) <10% of WBC Myeloblasts rarely observed Monocyte count <1 × 10^9^/L No dysgranulopoiesis
Hypercellular BM Neutrophil granulocytes increased in percentage and number Neutrophil maturation appears normal Myeloblasts <5% of nucleated cells
Not meeting WHO criteria for BCR-ABL1-positive CML, PV, ET, or PMF
No rearrangement of PDGFRA, PDGFRB, or FGFR1, or PCM1-JAK2
Presence of CSF3R T618I or other activating CSF3R mutation or in the absence of a CSFR3R mutation, persistent neutrophilia (at least 3 months), splenomegaly, and no identifiable cause of reactive neutrophilia including absence of a plasma cell neoplasm or, if present, demonstration of clonality of myeloid cells by cytogenetic or molecular studies
**Pre-PMF criteria** (diagnosis of pre-PMF requires meeting all three major criteria and at least one minor criterion)
Major criteria Megakaryocytic proliferation and atypia, without reticulin fibrosis >grade 1, accompanied by increased age-adjusted BM cellularity, granulocytic proliferation, and often decreased erythropoiesis Not meeting the WHO criteria for BCR-ABL1-positive CML, PV, ET, myelodysplastic syndromes, or other myeloid neoplasms Presence of JAK2, CALR, or MPL mutation or in the absence of these mutations, presence of another clonal marker, or absence of minor reactive BM reticulin fibrosis
Minor criteria Presence of at least one of the following, confirmed in two consecutive determinations: Anemia not attributed to a comorbid condition Leukocytosis ≥11 × 10^9^/L Palpable splenomegaly LDH increased to above upper normal limit of institutional reference range

The differential diagnosis between aCML and CNL was traditionally difficult since the proportion of immature myeloid cells (promyelocytes, myelocytes, and metamyelocytes) in PB (≥10% in aCML and <10% in CNL) and the presence of dysplasia were the only distinctive features (1, 17). Currently, the distinction between the two disorders is also supported by the high frequency of *CSF3R* mutations (42) in the latter and by the higher frequency of *SETBP1* and *ETNK1* mutations in the former. However, it is important to note that none of these variants are fully restricted to one of the two disorders (43), which suggests that they represent a continuum of related diseases rather than truly distinct entities.

On the other hand, CMML can be ruled out by the lack of monocytosis (1, 3), since in CMML, monocyte count must exceed 10% of the total leukocytes. Although effective, the not uncommon detection of borderline monocyte fractions at the onset occasionally renders the application of this hard threshold troublesome.

Finally, aCML diagnosis must not meet the WHO criteria for primary myelofibrosis (PMF), polycythemia vera (PV), or essential thrombocythemia (ET). In this context, the most challenging differential diagnosis is between aCML and pre-PMF, where the lack of an overt fibrosis and the common presence of myeloid leukocytosis with immature myeloid cells in PB of pre-PMF patients may render the differential diagnosis challenging. Luckily, the availability of the three myeloproliferative gene markers, *JAK2*, *CALR*, and *MPL*, allows to quickly discriminate between the two conditions. Conversely, the distinction between aCML and those rare myeloproliferative cases lacking all the three markers (i.e., the so-called triple-negative myeloproliferative disorders) is currently much more blurred.

## Prognosis

aCML is an aggressive disease generally associated with poor outcome, with a median overall survival (OS) of 11–25 months and a 5-year OS of 25% (4, 11, 18, 19, 43–47). Fifteen percent to 40% of aCML patients progress to secondary acute myeloid leukemia (sAML), with a median time to leukemic evolution of 11.2 months (11); the remaining patients usually develop complications related to BM failure. Unfavorable prognostic factors for OS are an increased WBC count (>50 × 10^9^/L) at presentation, increased immature precursors in the PB, age greater than 65 years at onset, hemoglobin (Hb) <10 g/dl, leukocyte count ≥50 × 10^9^/L, and immature circulating precursors (10, 11, 15, 43, 47). In addition, mutations in *ASXL1* and *SETBP1* have been associated with a more aggressive disease (10, 48), although their prognostic impact is still unclear (14). The risk of progression to sAML seems to be higher in case of palpable hepato- or splenomegaly, monocytosis, BM blastosis >5%, marked dyserythropoiesis, and transfusional requirement (11). In a recent work, stratification based on RNA-sequencing data identified two populations in terms of overall survival, and the overexpression of *DNPH1*, *GFI1B*, and *PARP1* genes has been correlated with poor prognosis (14).

## Genetics

The molecular features of aCML include an increased frequency of karyotypic abnormalities. In up to 80% of patients with aCML, additional chromosomal abnormalities (ACA) such as trisomy 8 or 9, del(20q), and −7/7q or isochromosomes 17q are the most common ones (4, 10, 11, 24, 49), but also aberrations involving chromosomes 12, 13, 14, 19, and 21 are reported (19, 44, 46, 50). Interestingly, trisomy 8, isochromosome 17q, and trisomy 19 are the most frequent anomalies observed in *BCR-ABL1*-positive CML, which are associated with blast crisis transformation (51–53).

The most frequent somatic mutations involve *SETBP1*, *ASXL1*, *NRAS*, *KRAS*, *ETNK1*, *SRSF2*, *EZH2*, *RUNX1*, and *TET2*, while mutations in *CBL*, *CSF3R*, and *JAK2* are less frequent (10, 18, 20, 22–24, 43, 54–60). Even though mutations occurring in *SETBP1* and *ETNK1* are not univocally disease-specific, they represent the alterations most closely associated with aCML (1, 22–24, 58). *SETBP1* and *ASXL1* are considered as high-risk mutations (43, 54), while *TET2* (61), *CBL* (62), and *EZH2 (* 63) mutations may suggest a possible overlap between aCML and CMML at the molecular level. On the other hand, the absence of rearrangements involving *PDGFRA* or *PDGFRB* (3) and *FGFR1* (1) and the negativity for *JAK2* V617F mutation (64) all support a diagnosis of aCML. The mutation frequency of the main aCML oncogenes, compared with *BCR-ABL1*-positive CML, AML, MDS, CMML, CNL, and MDS/MPN-U, is reported in **Table 1**.

## Molecular Landscape of aCML: Molecular Alterations and Associated Molecular Pathways

### SETBP1

**Figure 1:**
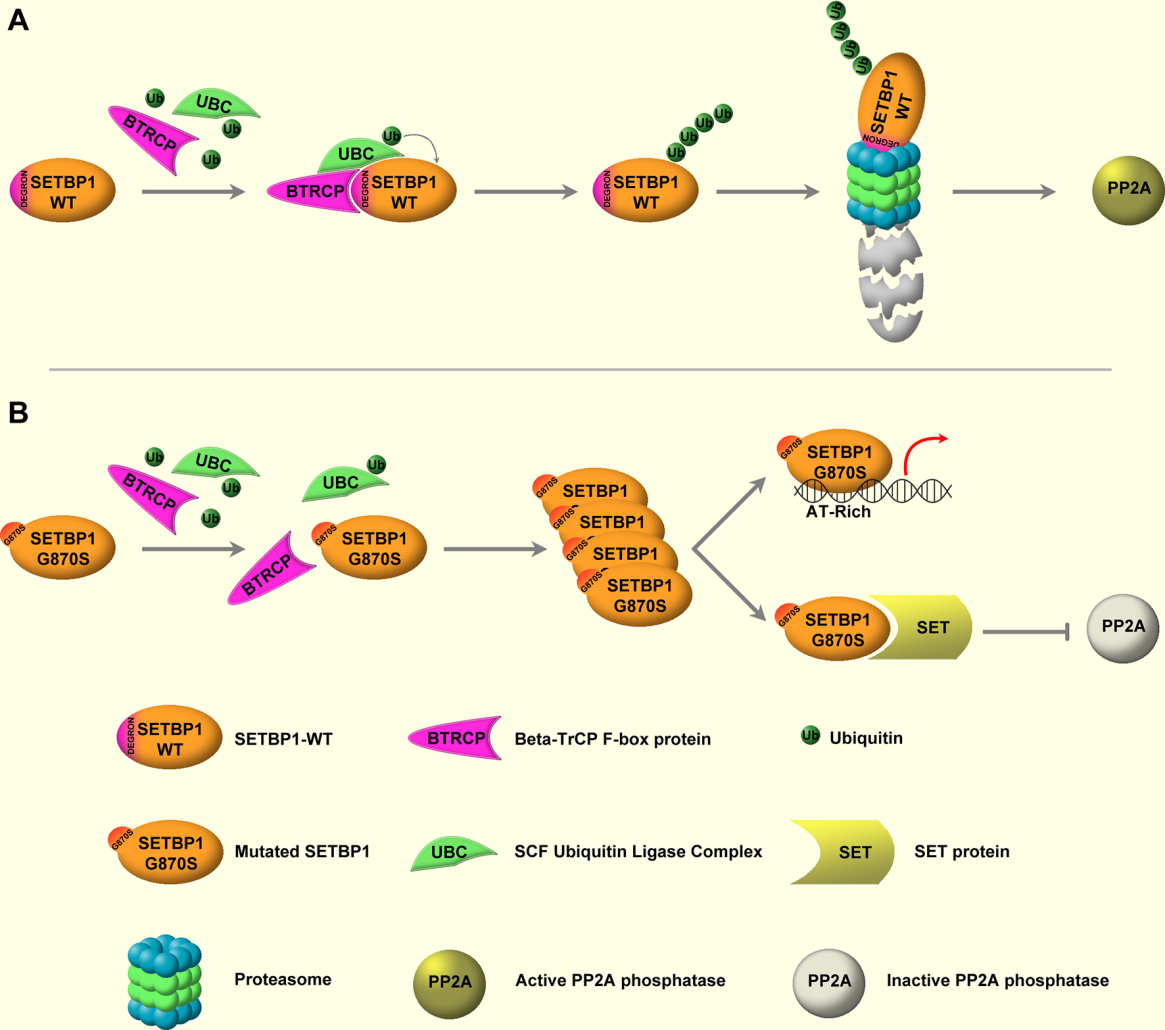
WT and mutated SETBP1 signaling. **(A)** SETBP1–WT is under the post-translational control of the ubiquitin protein ligase complex (UBC) known as SCF, which is mediated by the β-TrCP F-box protein. β-TrCP directly interacts with the SETBP1 degron motif, mediating the recruitment of SCF and the subsequent ubiquitination of SETBP1. Following ubiquitination, SETBP1 protein is degraded through the proteasome. **(B)** Degron mutations impair the interaction between SETBP1 and β-TrCP, protecting SETBP1 from proteasomal degradation and causing accumulation of the protein. This in turn leads to overactivation of the SETBP1 downstream signaling, specifically: 1) modulation of target genes through direct binding of SETBP1 to genomic DNA, mediated by its three AT-hook domains, and 2) activation of the SETBP1–SET axis, leading to SET accumulation and inhibition of PP2A phosphatase activity.

The molecular lesions responsible for the onset and progression of aCML were unknown until 2013, when, by applying NGS techniques, the presence of recurrent somatic mutations in *SETBP1* was described (22, 24, 59, 65–70). *SETBP1* mutations have been identified in about one-quarter of patients affected by aCML (22), but also in 10%–16% of MDS/MPN unclassifiable cases (24) and in 4%–15% of CMML patients (59). Moreover, *SETBP1* mutations have been occasionally described in juvenile myelomonocytic leukemia (JMML) and in about 1.7%–7% of sAML arising from MPN or MDS (57). Several studies had shown that *SETBP1* mutations are associated with an adverse clinical presentation, with a higher leukocyte count, a lower Hb level, and thrombocytopenia (22, 24, 71). These data suggest that this alteration is important not only for the dissection of the mechanisms of leukemogenesis, but also because it likely provides important prognostic value (72). *SETBP1* maps on chromosome 18q21.1 and encodes for SET-binding protein 1a, a protein of 1,596 amino acids with a predicted molecular weight of 170 kDa. The protein contains a SET-binding region and a SKI homology region, in which the recurrent mutations are clustered. The latter is highly conserved among vertebrates, suggesting an important but still unknown biological function. Moreover, three AT hooks can be found in SETBP1 protein and they are likely responsible for the direct interaction occurring between SETBP1 and the genomic DNA. It is known that SETBP1 is a binding partner for the SET nuclear oncoprotein (73). In turn, SET binds and negatively regulates the phosphatase 2A (PP2A) (74) oncosuppressor, a major phosphatase implicated in many cellular processes, such as cellular proliferation (75–79). In particular, PP2A loss of function has been associated with cell transformation (80, 81). Indeed, PP2A is a tumor suppressor that acts by regulating several signaling pathways critical for malignant transformation, such as AKT and ERK1/2 (82–84). By directly interacting with SET, SETBP1 protects it from proteolytic cleavage, increasing the amount of full-length SET protein and leading to the formation of a SETBP1–SET–PP2A complex resulting in PP2A inhibition, ultimately causing increased proliferation and expansion of the leukemic clone (85). We originally demonstrated that the majority of SETBP1 somatic mutations cluster in a mutational hotspot within the SKI-homologous region of the protein, conferring a proliferative advantage to the mutated cells (22). This hotspot is part of a degron motif recognized by the F-box protein β-TrCP, one of the four subunits of the ubiquitin protein ligase complex known as SCF. Under physiological conditions, this interaction stimulates SETBP1 ubiquitination and degradation through the proteasome (**Figure 1**, upper panel). SETBP1 degron mutations severely decrease the affinity of β-TrCP to SETBP1, leading to the accumulation of SETBP1 protein, promoting its overexpression, and triggering the stabilization of SET at the protein level. The consequence of these events is the inhibition of PP2A (**Figure 1**, bottom panel). Besides the interaction with the SET–PP2A axis, SETBP1 is also able to directly interact with genomic DNA through its three conserved AT hooks (86), recruiting transcriptional modulators such as HCF1, KMT2A, PHF8, and PHF6, which belong to the SET/KMT2A (MLL1) COMPASS-like complex, forming a multiprotein complex that in turn causes the activation of gene expression. Notably, SETBP1 binds to the promoter of *MECOM*, which modulates the expression of several genes involved in the proliferation of hematopoietic stem cells and in the myeloid differentiation, upregulating it (86, 87). SETBP1 overexpression also confers self-renewal capability to myeloid progenitors *in vitro* by interacting with the homeobox A9 (*HOXA9*) and homeobox A10 (*HOXA10*) promoters (88). It is also reported to interact with the *Runx1* promoter, resulting in Runx1 downregulation (89) and impairment of the Runx1-dependent program of myeloid differentiation (90, 91). Globally, these data suggest a complex role for SETBP1 as a transcriptional modulator, likely being able to activate or repress the expression of target genes depending on the coactivator/corepressor complexes co-recruited to the target locus.

### ETNK1

**Figure 2:**
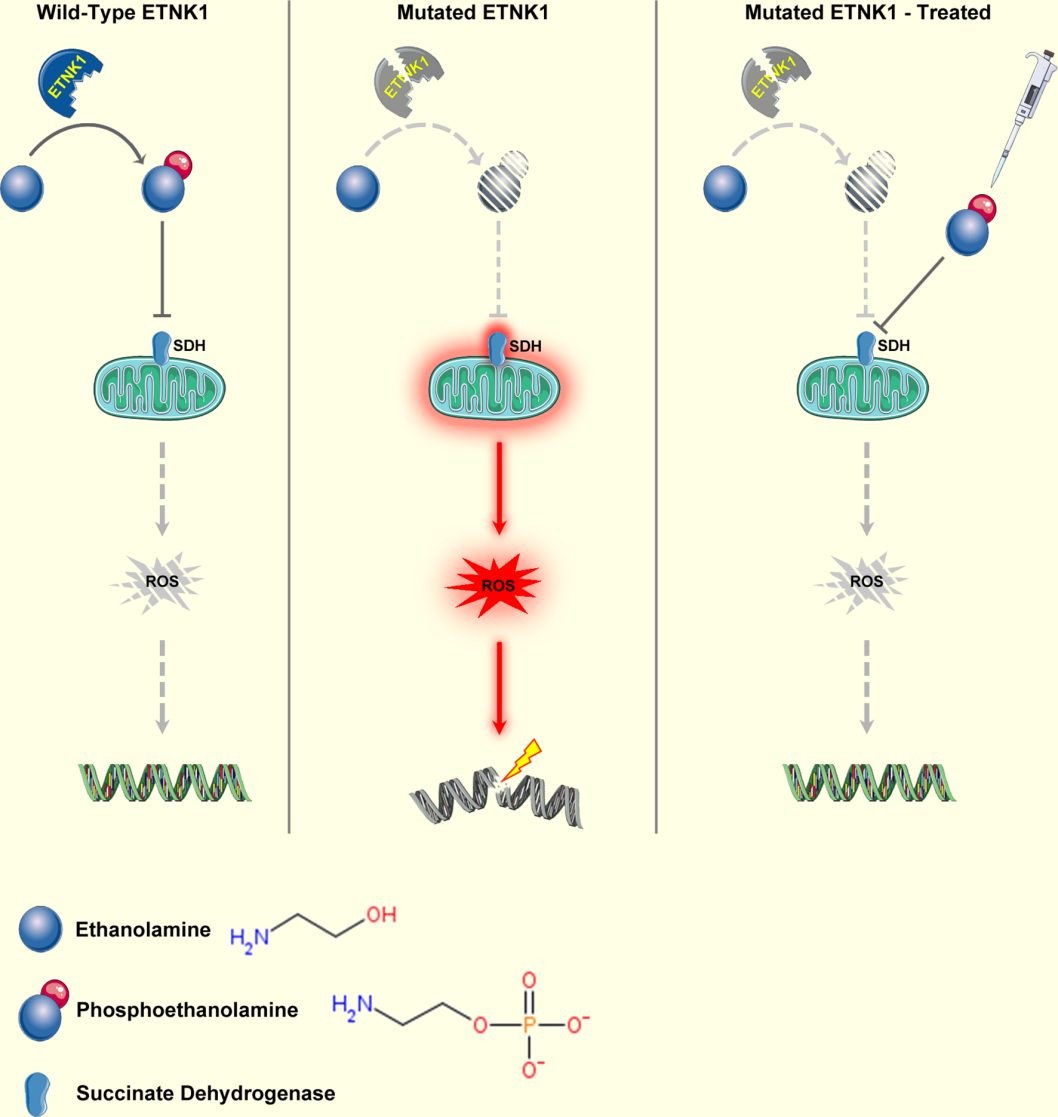
Mechanism of ETNK1 somatic mutations. Left panel: WT ETNK1 phosphorylates ethanolamine, causing the accumulation of phosphoethanolamine. Phosphoethanolamine directly inhibits mitochondrial complex II, also known as succinate dehydrogenase, therefore downmodulating mitochondrial activity and ROS production. Middle panel: mutated ETNK1 causes a decreased production of phosphoethanolamine, which in turn leads to an increased mitochondrial activation, increased ROS production, and DNA damage. Right panel: treatment of ETNK1-mutated cells with exogenous P-Et restores the normal mitochondrial activity through direct suppression of SDH activity, normalization of ROS production, and protection of DNA from ROS-mediated damage. The image was obtained from: *Nat Commun. 2020 Nov 23;11(1):5938* under a Creative Commons Attribution 4.0 International License. Elements of the image were obtained from https://smart.servier.com/under a Creative Commons Attribution 3.0 License.

The *ETNK1* gene (also known as *EKI1*) maps on chromosome 12p12.1. It spans 60.5 kb and consists of eight exons and seven introns (92). *ETNK1* encodes a protein of 452 residues known as ethanolamine kinase, a cytoplasmic enzyme that catalyzes the first step of the *de novo* phosphatidylethanolamine (PE) biosynthesis through the Kennedy or cytidine diphosphate (CDP)-ethanolamine pathway (93). The Kennedy pathway is responsible for the *de novo* biosynthesis of the two major membrane phospholipids, phosphatidylcholine (PC) and PE. In particular, ETNK1 is responsible for the phosphorylation of ethanolamine to generate phosphoethanolamine (P-Et) (93). Somatic *ETNK1* mutations were originally identified by our group in 13.3% of an aCML cohort (23). ETNK1 mutations were present as a heterozygous variant in the dominant clone and affected two contiguous residues: H243Y and N244S. Mutations clustering in the same hotspot of the kinase catalytic domain (N244S, N244T, N244K, G245A, G245V) were subsequently found also in 0%–14% of CMML cases (23, 94), in 20% of patients affected by aggressive systemic mastocytosis (SM) with eosinophilia (94), and in a single case of diffuse large B-cell lymphoma (DLBCL) (95). Recently, our group demonstrated that somatic *ETNK1* mutations are responsible for a reduced activity of the enzyme, causing a decrease in P-Et synthesis (23, 96). Through the reduced competition of P-Et with succinate at mitochondrial complex II, an increased mitochondrial hyperactivation is triggered, which in turn is responsible for increased ROS production and subsequent DNA damage and accumulation of further mutations (96). Treatment with exogenous P-Et is able to restore a normal phenotype, protecting cells from ROS-mediated DNA damage (96, 97) (**Figure 2**). Notably, recent findings suggest that, whenever present, *ETNK1* mutations occur at the initial stages of the clonal evolution of aCML (14), preceding other driver events, such as *ASXL1* or *SETBP1*, which indirectly supports the role of *ETNK1* as an inducer of a mutator phenotype.

### ASXL1

The *ASXL1* gene is located on chromosome 20q11.1, spanning 81 kb. This gene belongs to the polycomb gene family and plays a role in the recruitment of the Polycomb Repressor Complex 2 (PRC2) to its target sequences. It is also a component of the H2AK119 complex, responsible for histone H2A deubiquitination (98). *ASXL1* is mutated in more than 40% of aCML patients (14), and its mutations are associated with progression to acute phase and lower overall survival (22). It is currently known that ASXL1 contributes to the balance between the Polycomb Repressor Complex 1 (PRC1) and 2 (PRC2) in favor of the latter. Specifically, by interacting with the ubiquitin carboxy-terminal hydrolase BAP1, ASXL1 causes H2A Lysine 119 deubiquitination, therefore directly counteracting the activity of PRC1 (99). Instead, in combination with the PRC2, it promotes H3K27 trimethylation through the recruitment of the PRC2 effectors EZH1 and EZH2 at the target site, ultimately causing gene silencing.

*ASXL1* mutations are typically frameshift or nonsense mutations causing a C-terminal truncation of the ASXL1 protein. Constitutive as well as hematopoietic-lineage-restricted, homozygous ASXL1 knockout causes impairment of the bone marrow self-renewal capacity, ultimately leading to an MDS-like disease in mice (100, 101). In line with its role in promoting PRC2 activity, ASXL1 knockout confers a pan-reduction of the H3K27 trimethylation mark (27), leading to derepression of posterior *Hoxa* genes and oncogenic miR125a microRNAs. Importantly, overexpression of a truncated form of ASXL1, in combination with overexpression of BAP1, caused an important reduction in the global level of H3K27me3, together with a depletion of the H2AK119ub mark, therefore suggesting that the C-terminal truncation mutations may impair the PRC2 activity of ASXL1 while preserving its interaction with BAP1. Globally, *ASXL1* mutations act as loss-of-function events responsible for the promotion of myeloid transformation through loss of PRC2-mediated gene repression (27); however, their exact role is currently not entirely understood and likely multifaceted.

### TET2

The *TET2* gene maps on chromosome 4q24, spreading over 150 kb. TET2 is responsible for the modulation of DNA hydroxymethylation by converting 5-methylcytosine (5mC) to 5-hydroxymethylcytosine (5hmC) as the first step required to promote DNA demethylation (102, 103). Mutations occurring in *TET2* are present in about 30% of aCML cases (14, 104–106); therefore, they are among the most frequent mutations occurring in this disorder, together with *SETBP1* and *ASXL1*. *TET2* mutations are invariably linked to a global decrease in the 5hmC mark, which suggests that they represent loss-of-function events. This evidence is also corroborated by the non-focal pattern of these variants, as TET2 mutations can be found throughout the entire coding region of the gene, and further supported by the frequent occurrence of nonsense and frameshift events.

TET2 plays a critical role in the bone marrow, mostly by promoting hematopoietic stem cell differentiation (107). In line with this role, it is expressed at high levels in progenitor cells and its deletion causes an increase in immature progenitor cells (35, 108), promoting myeloid as well as lymphoid malignancies in mice (109). From a prognostic point of view, the role of TET2 mutations is not univocal, but their presence probably does not negatively impact on the overall survival in most hematological malignancies (36, 110). In the context of MDS/MPN, individual reports suggest that TET2 mutations may be detrimental (111); however, its accurate prognostic role remains to be ascertained.

### RAS

The *RAS* family of oncogenes comprises *HRAS*, *NRAS*, and *KRAS* genes. *HRAS* spans 3 kb and is located on chromosome 11p15.5, *NRAS* spans 7 kb and maps on chromosome 12p12.1, while *KRAS* spans more than 35 kb and is located at 1p13. These three genes share similar structures and sequences (112). The main product of the *RAS* genes is membrane-associated GTPases that control the MAP kinase cascade of serine/threonine kinases. Recurrent mutations in *RAS* genes occur in about 11%–27% of aCML patients (14, 22) and lead to a constitutively active expression of the protein. Usually aCML patients show *NRAS* or *KRAS* mutations (113, 114), and the most frequent mutations occur at codons 12, 31, and 61 (115–117).

### EZH2

The *EZH2* gene is located at 7q36.1 and encodes the histone methyltransferase representing the catalytic subunit of the PRC2. In particular, EZH2 methylates histone H3 at lysine-27 (H3-K27), promoting the epigenetic silencing of genes involved in cell fate decisions (118, 119). The pattern of *EZH2* mutations is particularly complex, as EZH2 variants can be both gain (GOF) as well as loss of function (LOF), with GOF mutations, such as EZH2-Y646X, frequently found in lymphoid malignancies and, in particular, in non-Hodgkin lymphoma (120) and solid, non-hematological tumors and LOF typically found in myeloid malignancies. In the context of aCML, LOF *EZH2* mutations are seen in about 19%–30% of cases (14). The functional effect of EZH2 GOF mutations is to aberrantly increase H3K27me3, promoting transcriptional repression (121), which impairs B-cell differentiation and leads to an increased number and size of germinal centers (122). In contrast, LOF EZH2 mutations cause suppression of the H3K27me3 mark, causing overexpression of *BCAT1* and leading to enhanced branched chain amino acid metabolism and activation of mTOR signaling (123). Association of *EZH2* LOF mutations with poor prognosis was demonstrated in myelodysplastic syndromes (124), while their prognostic role in other disorders such as AML or aCML is less clear (125, 126).

### RUNX1

The *RUNX1* gene, also known as *AML1*, is located at chromosome band 21q22.12 and encodes the alpha subunit of the core-binding factor (CBF) complex (127). This complex is responsible for the transcriptional modulation of critical factors involved in growth, survival, and differentiation processes; ribosome biogenesis; cell cycle regulation; and p53 and transforming growth factor β signaling pathways (128). RUNX1 contains a runt-homology domain (RHD), which is responsible for DNA binding and interaction with the heterodimeric partner CBFβ and a TAD domain characterized by the presence of motifs binding to a large number of activating and repressor proteins. RUNX1 is known to be involved in more than 50 different chromosomal translocations. The t(8;21) involving *RUNX1* and *RUNXT1*, the t(12;21) occurring in pediatric acute lymphoblastic leukemia and generating the ETV6–RUNX1 fusion, and the t(3;21) occurring in therapy-related AML and myelodysplastic syndrome and involving the *MECOM* oncogene are among those that are the most frequent. In the t(8;21), the persistence in the fusion of the RHD domain allows the binding of the protein to the normal RUNX1 gene targets. The presence of the RUNX1T1 fusion partner causes the recruitment of corepressors carrying deacetylase activity to the target promoters, therefore impairing the normal trans-activation and changing the function of the protein into a repressor, hence causing neomorphic activity (129).

Single-nucleotide somatic mutations are also commonly found in myeloid malignancies, such as AML and MDS. They typically occur in the RHD and, with a much lower frequency, in the TAD domain and can be mono- or biallelic. *RUNX1* mutations include missense, nonsense, frameshift, deletions, and splicing mutations (130). Mechanistically, mutations occurring in the RHD domain usually inactivate the protein, while mutations occurring downstream of the RHD domain typically confer a weak dominant negative activity to the mutant (131). These mutations are functionally distinct from the chromosomal translocations and usually confer a worse prognosis. Mutations involving this gene are present in about 11%–15% of aCML cases (14), but are also found in 10%–37% of CMML patients (132–134).

### SRSF2

The *SRSF2* gene is located on chromosome 17q25.1 and encodes a protein that plays a role in the splicing of primary mRNA (135, 136). This protein contains an RNA recognition motif that promotes spliceosome assembly at adjacent splice sites allowing the removal of introns from the primary transcripts (137). Moreover, it plays an active role in transcription and elongation and in coupling transcription and splicing processes (138, 139). Mutations in key factors of the spliceosome, such as *SRSF2*, *SF3B1*, *U2AF1*, and *ZRSR2*, occur in a large fraction of myelodysplastic syndromes (140).

SRSF2 contains an RNA-binding domain (RBD) responsible for the interaction with exonic splicing enhancers and an SR (serine-arginine rich) domain directly interacting with the other splicing ribonucleoproteins.

p.P95H is by far the most common mutation occurring in the *SRSF2* gene (141, 142). This mutation alters the RNA-binding affinity and specificity of the RBD domain, resulting in higher affinity for CCNG than to the standard GGNG motif, at least *in vitro* (143).

The frequency of *SRSF2* mutations in aCML is 14%–65% of cases (10, 14, 58, 144, 145). Although *SRSF2* mutations have been associated with worse survival outcomes in low-risk MDS patients (146), its prognostic role in aCML is currently unclear.

### CBL

*CBL* is located on chromosome 11q23.3; it contains 16 exons and spans more than 110 kb (147). This gene encodes a protein that acts as an E3 ubiquitin ligase, being required for targeting substrates for degradation by the proteasome. CBL plays both positive and negative regulatory roles in tyrosine kinase signaling transduction pathways. CBL can bind to activated signaling complexes recruiting downstream signal transduction components or can target receptors that in turn trigger internalization of the receptor/ligand complex, promoting recycling or proteasomal degradation in endosomes (148–151). Besides aCML, *CBL* has also been found mutated in 5%–19% cases of CMML patients (152, 153). Moreover, mutations of *CBL* are frequently associated with uniparental disomy at 11q (14, 154).

### CSF3R

The *CSF3R* gene is located at 1p34.3 and encodes the transmembrane receptor of the granulocyte colony-stimulating factor 3, which plays an essential role in the growth and differentiation of granulocytes (155, 156). Somatic *CSF3R* mutations are found in a large fraction (50%–80%) of patients affected by CNL (157, 158), and their presence is now one of the diagnostic criteria for CNL, according to the 2016 revision to the World Health Organization classification of myeloid neoplasms (1). In contrast, their association with aCML remains controversial. Although a single study reported *CSF3R* mutations to be frequent in aCML (157), several other works showed that *CSF3R* mutations are restricted to CNL and very rare in aCML (14, 22, 43, 58, 158). Currently, two types of CSF3R mutations are known: 1) extracellular domain/membrane proximal point mutations, such as the p.T618I variant, and 2) cytoplasmic truncation mutations. Mutations belonging to the first group result in a constitutive activation of the receptor, which occurs independently from the presence of the ligand. These mutations activate downstream JAK family tyrosine kinase pathways that drive the proliferation of neutrophil precursors, and are typically sensitive to JAK inhibitors. Truncation mutations instead interfere with receptor internalization and degradation, causing constitutive overexpression of CSF3R and ligand hypersensitivity, and show sensitivity to SRC kinase inhibitors (157).

## Gene Expression Profiling

To date, there are very few papers investigating the gene expression profile of aCML cases (13, 14, 25). Faisal and colleagues analyzed the mRNA expression of 26 aCML and 59 CMML cases, comparing them to a cohort of reference samples. Their analysis revealed a significant change in the expression levels of *SETBP1*, *CDKN2A*, *GATA2*, *MPL*, *TMEM14C*, *CSF3R*, and *FLT3* genes. The strongest differential expression effect was detectable in *FLT3* in CMML samples compared with aCML and references ones and in both *SETBP1* and *CSF3R* in aCML and CMML cases. These findings are in line with the mutation frequency of these genes in aCML and CMML (25). In the work of Zhang, RNA-sequencing was performed on 76 samples of aCML, CNL, CMML, and MDS/MPN unclassifiable (MDS/MPN-U). Gene expression signatures identified three main sample clusters, with different proportions of all diagnoses in each group, associated with prognostic markers (13). On the other hand, our group performed RNA-sequencing on a cohort of 43 aCML patients, and stratification based on gene expression profile identified two different populations in terms of overall survival. In this context, overexpression of three genes (*DNPH1*, *GFI1B*, and *PARP1*) was predictive of poor prognosis (14). Contrary to these disorders characterized by high heterogeneity, several profiling studies in *BCR-ABL1*-positive CML have been reported. In particular, expression profiling analyses revealed a different signature associated with the classical t(9;22)(q34;q11) translocation or with variant t(9;22) rearrangements (159); similarly, signature analysis predicted imatinib response or resistance (160–166).

## Therapy

No drug has so far proved to be effective and no established standards of care exist for the treatment of aCML (48, 54). Moreover, no consensus recommendations such as risk-based treatment algorithms exist to help clinicians in choosing between a watch-and-wait approach and initiation of therapy. During the last years, different therapeutic approaches have been proposed, but at present, allogeneic hematopoietic stem cell transplantation (allo-HSCT) remains the only potentially curative treatment option for aCML, even if only limited reports are available (167–171). However, allo-HSCT requires the presence of a suitable donor and is only available for young and middle-aged patients, since the toxicity of the transplant limits its use in the elderly (>70 years), where, especially with low-risk disease, monitoring or palliative chemotherapy may be more appropriate.

Regarding other medical therapies, different treatment strategies validated in other myeloid diseases have been evaluated in aCML. Hydroxyurea (HU) is used as a supportive care measure to control hyperleukocytosis and splenomegaly. Complete and partial hematological remissions have been reported in about 80% of patients, even if they are usually short-lived (10, 11, 19, 43, 44, 46, 47, 172–175). Moreover, complete and partial hematological remissions have also been reported after treatment with interferon alfa (IFN-alfa), even if many patients discontinued the treatment due to drug toxicity (19, 44, 172–174, 176, 177). However, both HU and IFN-alfa, despite being able to improve the WBC count, are unable to change the course of the disease and are typically used in a palliative setting where, due to the age of the patient or the presence of significant comorbidities, an allo-HSCT is not considered a valid option.

Among the other drugs that can be used in the treatment of aCML, especially in patients with high-risk disease, the hypomethylating agents (HMA), such as azacitidine or decitabine, are noteworthy (10, 47, 178–183). Indeed, based on their established activity in MDS and CMML, in which the overall response rates range from 25% to 70% and the overall survival from 12 to 37 months (184), these drugs could be used also in aCML. Nevertheless, up to now, the experience with HMA is still limited and the available data do not allow to accurately predict the efficacy of these compounds. Therefore, HMA cannot be considered as a standard of care for aCML yet and their use is off-label.

For selected patients with aggressive behavior, AML-like intensive chemotherapy is offered as a bridge to HSCT (185), even if this option has not been explored extensively.

Given the recent description of the mutational landscape of aCML, in the field of personalized therapies, different targeted drugs can be used for aCML treatment and are currently being investigated. Indeed, several actionable mutations have been identified in aCML which could be targeted, e.g., with tyrosine kinase inhibitors. Wang and colleagues administrated RAS, FLT3, MAPK, MYC, or AKT inhibitors to their patients (43). The MEK1/2 inhibitor trametinib, approved for malignant melanoma, has been used in *RAS*-mutated aCML (14, 186). Khanna and collaborators reported an aCML case with *NRAS* G12V mutation who experienced a notable response to trametinib with improvement in blood counts and 14 months of disease control (186), while another patient carrying *NRAS* G12D mutation treated with trametinib obtained a hematological response with blood cell count normalization and reduction of splenomegaly for 3 months (14).

For rare cases of aCML with JAK2 V617F mutation (62, 187, 188), ruxolitinib, a JAK2 inhibitor approved by the FDA in intermediate- to high-risk MF and PV intolerant or resistant to HU, can be used (189, 190). This drug is also effective in patients carrying *CSF3R* T618I mutation, since membrane proximal mutations result in JAK–STAT pathway activation (157), while truncation *CSF3R* mutations are reported to be sensitive to dasatinib (14, 157). An open-label, single-arm, phase II multicenter study evaluated the safety and efficacy of ruxolitinib in patients affected by CNL or aCML, regardless of their mutation status (191). Interestingly, 4/6 aCML patients carrying *CSF3R* mutations and 7/17 patients with *CSF3R* WT reached partial (PR) or complete responses (CR). PR was defined as >50% reduction of WBC, absolute neutrophil count (ANC), and granulocytic dysplasia and >25% reduction in spleen volume; CR was defined as normalization of WBC count and ANC, no evidence of granulocytic dysplasia, and normal spleen. A phase I, open-label study is currently ongoing to evaluate the safety and efficacy of TGR-1202, a PI3K-delta inhibitor, administered together with ruxolitinib in patients with MDS or MDS/MPN including aCML (ClinicalTrials.gov Identifier: NCT02493530).

Since the treatment of aCML remains a challenge, Gotlib proposed a new treatment algorithm (54), based on several decision nodes, including the potential candidacy for allogeneic hematopoietic stem cell transplantation, the results of myeloid mutation panel testing, the eligibility for enrollment in clinical trials, and the opportunity to adopt strategies used for MDS or MPN. Considering that two or more mutations in distinct genes often occur in aCML patients, combination therapies with different drugs could represent a promising approach. Since multiple actionable mutations are often present in various combinations in aCML patients, their enrollment in clinical trials should be considered whenever possible.

## Conclusion

aCML is a *BCR-ABL1*-negative hematological disease characterized by poor survival. The challenges in the management of this leukemia comprise both the diagnosis, due to the overlap of several genetic mutations among different MDS/MPN disorders, and the treatment choices, since at present no standards of care are available, except for bone marrow transplantation that is the only curative option for younger patients. The application of NGS techniques led to the discovery of new genes involved in the onset of the disease, which is allowing the introduction of personalized therapies for aCML patients. Further studies will be required to thoroughly assess the efficacy of these new treatments. Importantly, transcriptomic studies done at single-cell resolution may also unravel new targetable pathways that could increase the therapeutic options available for the treatment of this aggressive disorder.

## Author Contributions

DF: writing—original draft preparation. CG-P: funding acquisition, writing—review and editing, and supervision. RP: funding acquisition, writing—review and editing, and supervision. All authors contributed to the article and approved the submitted version.

## Conflict of Interest

The authors declare that the research was conducted in the absence of any commercial or financial relationships that could be construed as a potential conflict of interest.

## Publisher’s Note

All claims expressed in this article are solely those of the authors and do not necessarily represent those of their affiliated organizations, or those of the publisher, the editors and the reviewers. Any product that may be evaluated in this article, or claim that may be made by its manufacturer, is not guaranteed or endorsed by the publisher.
